# Influence of aspirin on aging skeletal muscle: Insights from a cross‐sectional cohort of septuagenarians

**DOI:** 10.14814/phy2.15669

**Published:** 2023-04-20

**Authors:** William A. Fountain, Masatoshi Naruse, W. Holmes Finch, Alex Claiborne, Scott W. Trappe, Todd A. Trappe

**Affiliations:** ^1^ Human Performance Laboratory Ball State University Muncie Indiana United States

**Keywords:** aging, aspirin, COX inhibitor, inflammation, skeletal muscle

## Abstract

Aspirin is one of the most commonly consumed cyclooxygenase (COX)‐inhibitors and anti‐inflammatory drugs and has been shown to block COX‐produced regulators of inflammation and aging skeletal muscle size. We used propensity score matching to compare skeletal muscle characteristics of individuals from the Health ABC study that did not consume aspirin or any other COX‐inhibiting drugs (non‐consumers, *n* = 497, 74 ± 3 year, 168 ± 9 cm, 75.1 ± 13.8 kg, 33.1 ± 7.4% body fat, 37% women, 34% black) to those that consumed aspirin daily (and not any other COX‐inhibiting drugs) and for at least 1 year (aspirin consumers, *n* = 515, 74 ± 3 year, 168 ± 9 cm, 76.2 ± 13.6 kg, 33.8 ± 7.1% body fat, 39% women, 30% black, average aspirin consumption: 6 year). Subjects were matched (*p* > 0.05) based on age, height, weight, % body fat, sex, and race (propensity scores: 0.33 ± 0.09 vs. 0.33 ± 0.09, *p* > 0.05). There was no difference between non‐consumers and aspirin consumers for computed tomography‐determined muscle size of the quadriceps (103.5 ± 0.9 vs. 104.9 ± 0.8 cm^2^, *p* > 0.05) or hamstrings (54.6 ± 0.5 vs. 54.9 ± 0.5 cm^2^, *p* > 0.05), or quadriceps muscle strength (111.1 ± 2.0 vs. 111.7 ± 2.0 Nm, *p* > 0.05). However, muscle attenuation (i.e., density) was higher in the aspirin consumers in the quadriceps (40.9 ± 0.3 vs. 44.4 ± 0.3 Hounsfield unit [HU], *p* < 0.05) and hamstrings (27.7 ± 0.4 vs. 33.2 ± 0.4 HU, *p* < 0.05). These cross sectional data suggest that chronic aspirin consumption does not influence age‐related skeletal muscle atrophy, but does influence skeletal muscle composition in septuagenarians. Prospective longitudinal investigations remain necessary to better understand the influence of chronic COX regulation on aging skeletal muscle health.

## Graphical abstract

Aspirin is one of the most commonly consumed cyclooxygenase (COX)‐inhibitors and anti‐inflammatory drugs and has been shown to block COX‐produced regulators of inflammation in human skeletal muscle. Considering the negative association between inflammation and muscle mass in older individuals, data from large scale observational studies of aging may provide insight into the role of aspirin in the control of age‐related skeletal muscle atrophy. These cross sectional data suggest that chronic aspirin consumption does not influence age‐related skeletal muscle atrophy, but does influence skeletal muscle composition in septuagenarians.

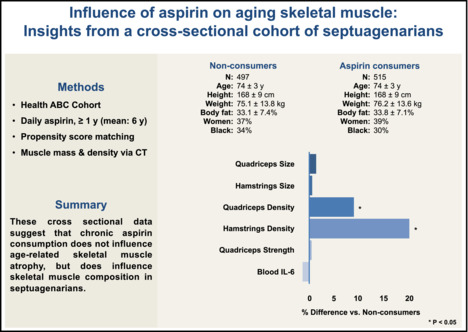

## 1 INTRODUCTION

Aging is associated with an elevated basal pro‐inflammatory status which can lead to concomitant reductions in skeletal muscle mass and function (Schaap et al., 2009; Visser, Pahor, et al., 2002). In addition to supporting ambulatory function and physical activity capacity, skeletal muscle mass is also important for its role in supporting the immune system (Nelke et al., 2019; Perkins et al., 2020). Interventions capable of reducing chronic inflammation in aging populations such as exercise training have been associated with numerous benefits in skeletal muscle tissue (Lavin et al., 2020; Mikkelsen et al., 2013; Petersen & Pedersen, 2005). Interestingly, regular anti‐inflammatory drug consumption has been associated with a lower incidence of sarcopenia in healthy octogenarians (Landi et al., 2013), but there are relatively few studies in this area that focus on skeletal muscle and our overall understanding is limited (Alturki et al., 2018; Espinoza et al., 2022; Orkaby et al., 2021, 2022; Trappe & Liu, 2013).

Aspirin is perhaps the most common anti‐inflammatory drug since it is available over the counter and often taken daily for its cardioprotective effects (Ansa et al., 2019; McNeil, Wolfe, et al., 2018; Stuntz & Bernstein, 2017). The cyclooxygenase (COX) pathway is an established component of inflammation‐associated regulatory mechanisms in various tissues including skeletal muscle (Korotkova & Lundberg, 2014; Trappe & Liu, 2013). Aspirin has been shown to be a potent COX inhibitor in human skeletal muscle, even at low doses (Fountain et al., 2020; Naruse et al., 2021; Ratchford et al., 2017). Inhibition of the COX pathway in skeletal muscle alters prostaglandin production, muscle protein turnover, inflammation associated pathways, and ultimately has been shown to influence skeletal muscle mass (Trappe et al., 2016; Trappe & Liu, 2013).

The Health, Aging and Body Composition (Health ABC) Study examined skeletal muscle size using gold‐standard measurements (i.e., computed tomography, CT) in a large and diverse aging population. Previous reports from Health ABC have indicated a large prevalence of anti‐inflammatory drug use in this cohort (Carbone et al., 2003; Schaap et al., 2009; Visser, Pahor, et al., 2002). Therefore, the aim of the current investigation was to evaluate whether Health ABC participants with a history of long‐term aspirin consumption had larger skeletal muscle (quadriceps and hamstrings) size compared with non‐aspirin consumers. The CT measurement also allowed for an assessment of skeletal muscle attenuation, a non‐invasive measure of skeletal muscle composition (Goodpaster et al., 2001, 2022). To parallel the muscle size measurements, muscle strength was also assessed. Finally, systemic circulating levels of interleukin (IL)‐6 were examined as this pleiotropic cytokine has been shown to be involved in the regulation of skeletal muscle mass (Fountain et al., 2023).

## 2 METHODS

To be eligible for the Health ABC Study, participants had to report no difficulty walking at least one quarter mile and climbing 10 stairs without resting, no difficulty performing basic activities of daily living, no need for a cane, walker, crutches, or other assistive ambulatory equipment, no history of cancer treatment in the preceding 3 years, and no plan to move out of the area in the next 3 years. Health ABC aimed to recruit participants who were between 70 and 79 years of age resulting in an initial cohort of 3075 Black and White men and women. Participants in this cohort were recruited from Pittsburgh, Pennsylvania (*n* = 1527), and Memphis, Tennessee (*n* = 1548). Baseline information regarding demographics, medical history, and current health status was collected during an in‐home interview between April 1997 and May 1998. Annual clinic visits included anthropometric measurements, a comprehensive inventory of current medications and supplements, and evaluation of body composition using CT and dual energy x‐ray absorptiometry (DXA). If a participant did not have valid CT data or were missing records of their medication use, they were excluded from the current investigation. All participants signed an informed written consent which was approved by the Institutional Review Boards of the University of Pittsburgh and the University of Tennessee in accordance with the Declaration of Helsinki. The current investigation was also approved by the Institutional Review Board at Ball State University.

### 2.1 Medication inventory

To complete the medication inventory, participants were asked to provide all medications and supplements they used within the past 2 weeks. Drug names and consumption frequency (daily, weekly, or monthly) were recorded. Participants were also asked when they began taking each drug. Drug names were matched with the Iowa drug information service (IDIS), a hierarchical coding system which categorizes pharmaceutical ingredients (Pahor et al., 1994). The IDIS classification of “analgesics & antipyretics” consists of several sub‐categories (NSAIDs, celecoxib, salicylates, opioids, other) grouping over 30 drugs with various intended uses and mechanisms of action.

The most common mechanism of action of drugs in this class is inhibition of the COX pathway and several of these drugs have been shown to reduce human skeletal muscle COX pathway activity (Boushel et al., 2002; Burian et al., 2013; Fountain et al., 2020; Naruse et al., 2021; Ratchford et al., 2017; Trappe et al., 2001). Aspirin (IDIS: 28080751) was the most prevalent COX‐inhibitor in Health ABC, followed by acetaminophen (IDIS: 28081221), ibuprofen (IDIS: 28080509), naproxen (IDIS: 28080513), nabumetone (IDIS: 28080459), etodolac (IDIS: 28080455), diclofenac (IDIS: 28080422), sulindac (IDIS: 28080428), indomethacin (IDIS: 28080463), ketoprofen (IDIS: 28080512), oxaprozin (IDIS: 28080466), meclofenamate sodium (IDIS: 28080412), tolmetin (IDIS: 28080420), piroxicam (IDIS: 28080437), fenoprofen (IDIS: 28080506), flurbiprofen (IDIS: 28080507), celecoxib (IDIS: 28080601), choline salicylate (IDIS: 28080752), magnesium salicylate (IDIS: 28080756), phenyl salicylate (IDIS: 28080761), salsalate (IDIS: 28080764), diflusinal (IDIS: 28080768), and ketorolac (IDIS: 28081219).

### 2.2 Skeletal muscle size, attenuation, and strength

Cross‐sectional area (CSA, cm^2^) and attenuation of the mid‐thigh muscles were measured by CT (Memphis site: Siemens Somatom Plus 4 [Siemens] and Picker PQ 2000S [Marconi Medical Systems], Pittsburgh site: GE 9800 Advantage [General Electric]) as previously described in detail (Goodpaster et al., 2001; Visser, Kritchevsky, et al., 2002; Visser, Pahor, et al., 2002). An anterior–posterior scout scan of the entire femur was used to localize the mid‐thigh. The femoral length was measured in the cranial‐caudal direction and the midpoint of the distance between the medial edge of the greater trochanter and the intercondyloid fossa was used for the scan location. A single, 10‐mm thick axial image of each thigh was analyzed using IDL development software (RSI Systems) for muscle CSA and muscle attenuation. CT numbers were defined on the Hounsfield unit (HU) scale where 0 corresponds with the density of water and −1000 corresponds with air. The external contours of the thigh were determined using a threshold of −224 HU, and the external bone contours were derived at 150 HU. For each participant, skeletal muscle and adipose tissue were differentiated using a bimodal image distribution histogram resulting from the distribution of CT numbers in adipose tissue and muscle tissue in the entire image (Seidell et al., 1987). Intermuscular and visible intramuscular adipose tissue was separated from subcutaneous adipose tissue by drawing a line along the deep fascial plane surrounding the thigh muscles. Muscle borders that were not already defined by adipose tissue were manually outlined. The quadriceps (rectus femoris, vastus lateralis, vastus medialis, vastus intermedius) and hamstrings (biceps femoris short and long heads, semitendinosus, semimembranosus) were manually outlined for each participant. The mean muscle area was calculated by multiplying the number of pixels of nonbone, nonadipose tissue by the pixel area. Skeletal muscle area for each muscle group in both legs was added together to provide total CSA of the quadriceps and hamstrings. Mean muscle attenuation was calculated by averaging the CT number (pixel intensity) values of the regions outlined on the images. Muscle attenuation from left and right muscles was determined separately and averaged for each muscle with the area of each side considered. Maximal isokinetic (60°/s) strength was determined unilaterally on a dynamometer as described previously (Goodpaster et al., 2001).

### 2.3 Cytokine IL‐6

Plasma concentration of IL‐6 was determined from blood samples collected in the morning after an overnight fast of at least 8 h. Sample processing and assay details have been described previously (Visser, Pahor, et al., 2002).

### 2.4 Participants and propensity score matching

**FIGURE 1:**
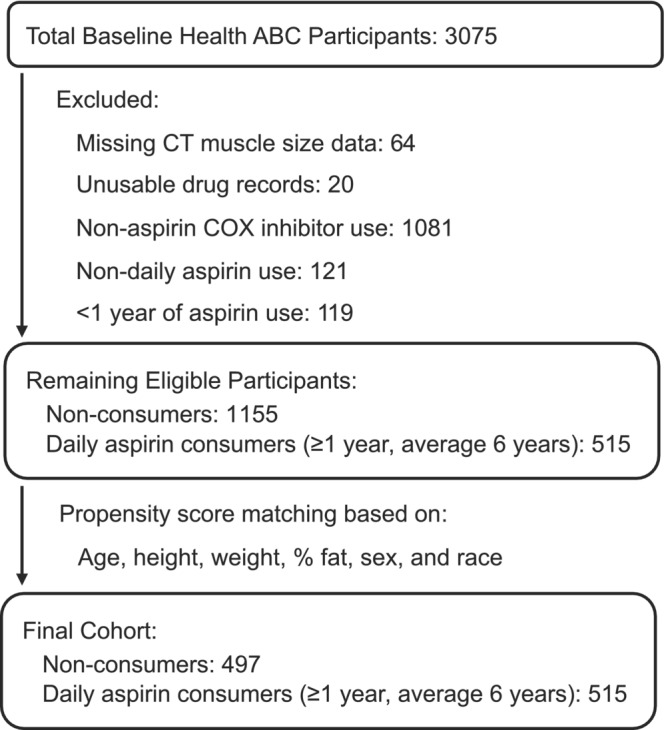
Participant exclusions and propensity score matching used to produce the final cohort used in the current investigation. ABC, Aging and Body Composition; CT, computed tomography; COX, cyclooxygenase.

Participants who (1) consumed aspirin on a daily basis, (2) consumed aspirin for at least 1 year leading up to their CT scan for muscle mass determination and (3) did not consume any other COX‐inhibiting drug (daily, weekly, or monthly) were classified as aspirin consumers (*n* = 515). Participants who did not consume aspirin or any other COX‐inhibiting drug (daily, weekly, or monthly) were classified as non‐consumers (*n* = 1155). These groups of aspirin consumers and non‐consumers were then considered eligible for propensity score matching (Figure 1).

Propensity score matching (Austin, 2009) was completed after generating propensity scores for each individual using logistic regression considering age, height, weight, % body fat, sex, and race as predictor variables (Table 1). Within study site propensity score matching was completed first and then combined to form the final matched cohorts (Chang & Stuart, 2022). This approach was completed because of a significant difference in quadriceps and hamstrings muscle size between the two study sites in the original cohort and the sub‐cohort used in the current investigation. Without this approach, propensity score matching resulted in a substantial study site imbalance between the groups, even when study site was also considered in the logistic regression propensity score generation and subsequent matching. The success of the matching was confirmed with (1) no statistical difference in any of the matching variables, (2) the low standardized differences (<10%) for all of the matching variables (Austin, 2009), and (3) the similarity of propensity scores between non‐consumers and aspirin consumers (Table 1).

**TABLE 1:** Participant characteristics.

	Non‐consumers	Aspirin consumers	*p*	Standardized difference, %
*n*	497	515		
Age, year	74 ± 3	74 ± 3	0.41	5.2
Height, cm	168 ± 9	168 ± 9	0.83	1.3
Weight, kg	75.1 ± 13.8	76.2 ± 13.6	0.19	8.2
% Body fat	33.1 ± 7.4	33.8 ± 7.1	0.12	9.8
% Women	37	39	0.55	3.7
% Black	34	30	0.23	7.5
% Pittsburgh	56	56	0.85	1.2
Propensity score	0.33 ± 0.09	0.33 ± 0.09	0.49	—

*Note*: Data are mean ± SD.

### 2.5 Statistics

Propensity score matching and statistical analyses were performed using SPSS (Version 28.0; IBM Corp). Matching variables between groups were compared with a *t*‐test or chi‐squared test. Statistical comparisons of skeletal muscle size, attenuation, and strength, as well as plasma IL‐6 levels (log‐transformed) between groups were made using a three‐way analysis of covariance (ANCOVA; sex, race, aspirin) controlling for site. Because chronic daily aspirin consumption is widely used for prevention of cardiovascular disease (CVD; Ansa et al., 2019; McNeil, Wolfe, et al., 2018; Stuntz & Bernstein, 2017; defined in Health ABC as coronary heart disease and cerebrovascular disease), CVD was also included with site as a covariate in an additional comparison. The participant characteristics are presented as mean ± SD and the skeletal muscle characteristics are presented as least‐square (LS) mean ± SE. Significance was accepted at *p* < 0.05.

## 3 RESULTS

**FIGURE 2:**
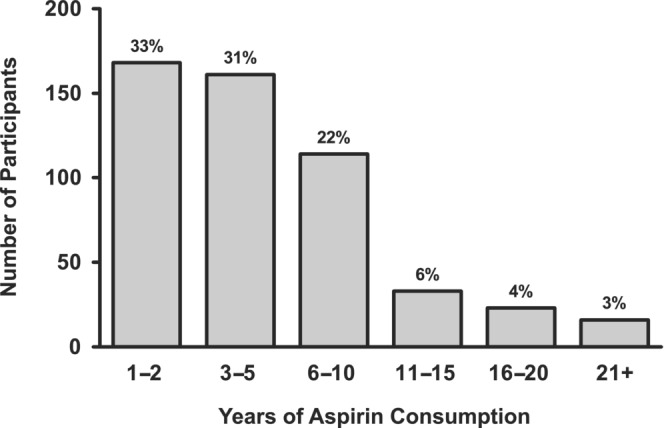
Duration of aspirin use in the aspirin consumers group. Average aspirin consumption duration was 6 years.

While the original goal was simply to capture participants with at least 1 year of aspirin consumption, the aspirin consuming cohort had a much longer average duration of aspirin use (6 year). In fact, approximately one‐third of participants (35%) had been consuming aspirin for six or more years (Figure 2). The absolute daily aspirin dose (mg/day) could not be verified for every participant; however, the extended duration of use would suggest low‐dose consumption.

There were differences (*p* < 0.05) between men and women, and White and Black participants for quadriceps (men: 124.1 ± 0.7, women: 84.3 ± 0.9; White: 98.3 ± 0.7, Black: 110.1 ± 1.0 cm^2^) and hamstrings (men: 63.3 ± 0.4, women: 46.1 ± 0.6; White: 50.4 ± 0.4, Black 59.1 ± 0.6 cm^2^) skeletal muscle size, as has been previously shown in the larger Health ABC cohort (Goodpaster et al., 2001; Newman et al., 2003; Schaap et al., 2009; Taaffe et al., 2001; Visser, Kritchevsky, et al., 2002; Visser, Pahor, et al., 2002). Quadriceps and hamstrings skeletal muscle size, quadriceps skeletal muscle strength, and plasma IL‐6 levels were not different (*p* > 0.05) between the non‐consumers and aspirin consumers (Table 2). Quadriceps and hamstrings skeletal muscle attenuation were different (*p* < 0.05) between the non‐consumers and aspirin consumers (Table 2).

**TABLE 2:** Skeletal muscle characteristics and blood cytokine levels.

	Non‐consumers	Aspirin consumers
Skeletal muscle		
Size, cm^2^		
Quadriceps	103.5 ± 0.9	104.9 ± 0.8
Hamstrings	54.6 ± 0.5	54.9 ± 0.5
Attenuation, Hounsfield unit		
Quadriceps	40.9 ± 0.3	44.4 ± 0.3*
Hamstrings	27.7 ± 0.4	33.2 ± 0.4*
Strength, Nm		
Quadriceps	111.2 ± 2.0	111.7 ± 2.0
Blood		
IL‐6, pg/mL	2.1 (1.4–3.4)	2.1 (1.4–3.0)

*Note*: Skeletal muscle data are site‐adjusted least‐square mean ± SE. Interleukin 6 (IL‐6) data are median and (interquartile range). Size data reflect the sum of both legs, attenuation data reflect the average of both legs, strength data reflect one leg. For the IL‐6 data, log transformed data were analyzed (Visser, Pahor, et al., 2002).

* *p* < 0.05 non‐consumers versus aspirin consumers.

There was also no difference (*p* > 0.05) between the non‐consumers and aspirin consumers for quadriceps (103.2 ± 0.9 vs. 105.2 ± 0.9 cm^2^) and hamstrings (54.5 ± 0.5 vs. 54.9 ± 0.5 cm^2^) skeletal muscle size when CVD was considered. The results were also unchanged for skeletal muscle attenuation and strength, and IL‐6 levels when CVD was considered. There were no significant (*p* > 0.05) interactions between aspirin and the other main factors (sex and race) in any of the ANCOVAs for these variables.

## 4 DISCUSSION

This investigation, through the large and diverse cohort provided by Health ABC, offers the first large‐scale insight into the potential influence of chronic COX inhibition via aspirin on direct measurements of skeletal muscle size in older individuals. The lack of influence of aspirin on skeletal muscle size of two important upper leg muscle groups between non‐consumers and aspirin consumers was interesting considering the known effect of aspirin on the COX pathway in human skeletal muscle (Fountain et al., 2020; Naruse et al., 2021; Ratchford et al., 2017). In agreement with these muscle size findings was the lack of aspirin influence on muscle strength and circulating IL‐6. An unexpected finding was the influence of aspirin on skeletal muscle attenuation, which is associated with skeletal muscle lipid content and metabolic health (Goodpaster et al., 2022).

Aspirin uniquely inhibits the COX enzyme through an irreversible acetylation of the COX active site (Simmons et al., 2004). Thus, new COX protein production is required to overcome this inhibition, effectively extending or enhancing the potency of aspirin depending on the rate of tissue COX protein turnover. The COX pathway is one of many integrated regulatory mechanisms of skeletal muscle inflammation and several of its components are differentially expressed with advanced age (Lavin et al., 2020; Liu et al., 2016). COX enzyme activity is dependent upon the metabolism of arachidonic acid, an omega‐6 fatty acid that yields metabolites which have been identified as major contributors to the development of muscle weakness, atrophy, and disease (Korotkova & Lundberg, 2014). In skeletal muscle, prostaglandins (PGs) are the most abundant metabolite of arachidonic acid and are involved in the regulation of oxidative stress, blood flow, and muscle protein turnover (Boushel et al., 2002; Smith et al., 2011; Standley et al., 2013; Trappe & Liu, 2013). PGE_2_ is the most abundant PG produced by the COX pathway in skeletal muscle and when human skeletal muscle is incubated with PGE_2,_ it stimulates transcription of muscle RING finger‐1 (MuRF‐1), a ubiquitin ligase contributing to muscle proteolysis, and the pleiotropic cytokine IL‐6 (Standley et al., 2013).

The COX/PGE_2_/IL‐6 pathway has been connected to reduced muscle mass through mechanistic human studies, epidemiological findings, and preclinical animal models. IL‐6 infusion in young healthy volunteers resulted in a net loss of amino acids from skeletal muscle tissue, which was due to protein synthesis being blunted more than protein breakdown (van Hall et al., 2008). In addition, an inverse relationship between circulating IL‐6 concentrations and skeletal muscle protein synthesis rates in young and old men and women has been reported (Toth et al., 2005). Large‐scale studies of aging individuals, including Health ABC, also support the negative association of elevated systemic IL‐6 with muscle size and function (Cesari et al., 2004; Ferrucci et al., 2002; Schaap et al., 2009; Visser, Pahor, et al., 2002). In rat models, low level IL‐6 infusion into skeletal muscle induced muscle atrophy (Haddad et al., 2005) and reduced muscle growth (Bodell et al., 2009). In addition, 5 months of COX inhibition with ibuprofen treatment in old rats resulted in a 60% reduction in circulating IL‐6, increased muscle protein synthesis, reduced muscle proteolysis, and increased muscle mass (Rieu et al., 2009).

The disconnect between the aforementioned literature and the current findings regarding skeletal muscle mass is not readily apparent. We can speculate that aspirin (or other potential interventions) may not be as effective for older individuals (>70 year), like the individuals in Health ABC, because they were on the steepest part of the aging and skeletal muscle atrophy curve, at least as it has been defined for the quadriceps (Frontera et al., 2000; McPhee et al., 2018; Mitchell et al., 2012). It is also possible the inflammatory burden in skeletal muscle and other tissues at this age might outweigh the daily aspirin dose. In addition, redundant inflammatory mechanisms may override the COX pathway contribution, even if aspirin were effectively inhibiting COX. We also have to consider that the daily dose consumed by these individuals was too low to have a significant impact on COX activity in peripheral tissues like skeletal muscle, although the muscle attenuation findings might suggest otherwise. While we do not specifically know the dose consumed by each subject, which is a limitation of the current investigation, we assume chronic daily aspirin consumption would only be tolerated at what would be considered “low” dose (≤325 mg; Cryer & Feldman, 1998). A single oral dose of 975 mg has been shown to significantly suppress COX‐generated inflammatory regulators in human skeletal muscle by an average of 44%, and up to 97% in some individuals (Ratchford et al., 2017). However, lower doses have not been tested in vivo (Fountain et al., 2020; Naruse et al., 2021). The cross‐sectional design of the current investigation is a potential limitation, as studying the same individuals over time would provide a more robust approach to our research question. The observational design of the Health ABC study is also somewhat of a limitation, as a randomized controlled trial of aspirin consumption and direct measurements of skeletal muscle size would be more ideal. Our propensity score matching approach and well‐balanced groups should have helped minimize these last two issues.

The skeletal muscle attenuation findings were surprising and suggest a potentially altered tissue composition of the quadriceps and hamstrings muscles in the aspirin consumers. Studies using non‐invasive imaging like that used in the current investigation have suggested the attenuation levels reflect lipid infiltration of the muscle, which is associated with altered skeletal muscle metabolic health (Goodpaster et al., 2001, 2022). While the difference in attenuation observed between the two groups may reflect differences in metabolic function, it did not appear to reflect any difference in function at the level of muscle force generation. This is not surprising given the similarities in muscle size between the two groups, as well as the fact that muscle attenuation has been only weakly associated with muscle strength (Goodpaster et al., 2001). It is also interesting to note the hamstrings muscles have a lower attenuation value (i.e., more fatty infiltration) compared to the quadriceps (Table 2), and the difference in attenuation values between the non‐consumers and aspirin consumers was greater in the hamstrings (+20%) compared to the quadriceps (+9%). Differences in skeletal muscle attenuation have been reported across several muscles in the Health ABC cohort (Goodpaster et al., 2001; Naruse et al., 2023). Overall, these attenuation findings do suggest some influence of chronic aspirin consumption on skeletal muscle of older individuals that needs to be investigated further.

Other large trials investigating the influence of chronic low dose aspirin consumption provide some data to consider, although the primary outcomes of these studies did not focus on or make direct measurements of skeletal muscle (McNeil, Woods, et al., 2018; Steering Committee of the Physicains' Health Study Research Group, 1989). The Physicians' Health Study (PHS) and the ASPirin in Reducing Events in the Elderly (ASPREE) trial also reported findings related to the influence of aspirin on frailty (Espinoza et al., 2022; Orkaby et al., 2021) and walking speed (Orkaby et al., 2022). While frailty is a multi‐factorial condition (Espinoza et al., 2022; Fried et al., 2001), reduced skeletal muscle mass contributes to increased frailty and to lower walking speed. Orkaby et al. (2021, 2022) showed in the PHS cohort chronic aspirin consumption was associated with a lower prevalence of frailty and increased walking speed. This would suggest that aspirin may have had an influence on muscle mass. However, Espinoza et al. (2022) did not report any association between chronic aspirin consumption and frailty in the ASPREE cohort. Authors from both of these trials (Espinoza et al., 2022; Orkaby et al., 2021) suggested the relatively younger enrollment age in the PHS cohort compared to the ASPREE cohort may have contributed to the difference between the studies. Espinoza et al. (2022) also showed in a subgroup analysis of the older adults in the ASPREE trial that were consuming aspirin prior to the study (i.e., started aspirin relatively younger) did have a 25% reduction in frailty incidence. In general, these findings would align with our previous suggestions that the higher rate of skeletal muscle atrophy and/or a higher inflammatory burden in the older aging population may limit the effectiveness of the low dose aspirin. Additionally, the PHS trial only studied men while the ASPREE trial had 56% women. Aspirin inhibition of human skeletal muscle COX has been shown to be 60% greater in men compared to women (Naruse et al., 2021), which may also contribute to the difference in findings from these two large scale trials.

The influence of chronic inflammation in skeletal muscle and other tissues is inherently complex and controlling it remains a focus for improving the health and quality of life of aging individuals. Prospective studies remain necessary to better understand the regulatory influence of the COX pathway on inflammation and aging skeletal muscle health. These studies should consider pharmacological agents (e.g., aspirin) and other interventions (e.g., exercise) alone or in combination. Measurements should span from whole muscle and myocellular size and function to the underlying metabolic and cellular regulation while considering sex, race, various ages, and different muscle groups.

## AUTHOR CONTRIBUTIONS

Conception and design of research: William A. Fountain, Masatoshi Naruse, W. Holmes Finch, Alex Claiborne, Scott W. Trappe, Todd A. Trappe. Analyzed data: William A. Fountain, Masatoshi Naruse, W. Holmes Finch, Todd A. Trappe. Interpreted results of experiments: William A. Fountain, Masatoshi Naruse, W. Holmes Finch, Todd A. Trappe. Prepared figures: William A. Fountain, Masatoshi Naruse, Todd A. Trappe. Drafted manuscript: William A. Fountain, Masatoshi Naruse, Alex Claiborne, Todd A. Trappe. Edited and revised manuscript: William A. Fountain, Masatoshi Naruse, W. Holmes Finch, Alex Claiborne, Scott W. Trappe, Todd A. Trappe. Approved final version of manuscript: William A. Fountain, Masatoshi Naruse, W. Holmes Finch, Alex Claiborne, Scott W. Trappe, Todd A. Trappe.

## FUNDING INFORMATION

This research was supported by National Institute on Aging (NIA) Contracts N01‐AG‐6‐2101; N01‐AG‐6‐2103; N01‐AG‐6‐2106; NIA grant R01‐AG028050, and NINR grant R01‐NR012459. This research was funded in part by the Intramural Research Program of the NIH, National Institute on Aging.

## CONFLICT OF INTEREST STATEMENT

No conflicts of interest, financial or otherwise, are declared by the authors.
